# Self-multilayered cell sheet generated by dental pulp stem cells provides a mechanically quantifiable regenerative graft for bone tissue engineering

**DOI:** 10.1186/s13287-026-05304-x

**Published:** 2026-09-22

**Authors:** Mai Mochizuki, Taka Nakahara

**Affiliations:** 1 Department of Life Science Dentistry, The Nippon Dental University, 1-9-20 Fujimi, Chiyoda-ku, Tokyo, 102-8159 Japan; 2 Department of Developmental and Regenerative Dentistry, The Nippon Dental University School of Life Dentistry at Tokyo, 1-9-20 Fujimi, Chiyoda-ku, Tokyo, 102-8159 Japan

**Keywords:** Dental pulp stem cells, Self-multilayered cell sheet, Self-multilayering, Self-organization, 2D-to-3D transition, Extracellular matrix, Scaffold-free tissue engineering, Cell sheet engineering, Puncture-force assay, Bone tissue engineering

## Abstract

**Background:**

Mesenchymal stromal cell (MSC) sheets are promising regenerative grafts, but fragile scaffold-free sheets remain difficult to harvest, manipulate, and prepare as mechanically reliable grafts for transplantation. We found that human dental pulp stem cells (DPSCs) cultured on type I collagen-coated cultureware in xenogeneic serum-free medium (COL-XFM) autonomously generated a thick, handleable multilayered tissue without manual stacking or exogenous scaffold support. Here, we define this tissue as a DPSC-derived *self-multilayered cell sheet* (SMCS) and examine its formation, mechanical properties, and graft-organizing function.

**Methods:**

SMCS formation was optimized by seeding-density analysis under COL-XFM culture. The DPSC-optimized condition was then used to compare DPSCs with bone marrow-derived MSCs (BMSCs) and umbilical cord-derived MSCs (UCSCs). We developed a simple surface-contact puncture-force assay to quantify localized rupture resistance during sheet puncture. The day 8 (D8)–day 10 (D10) transition from a two-dimensional (2D) monolayer to three-dimensional (3D) SMCS was examined by analyzing type I collagen (COL1) production, TGF-β signaling, integrin α2 expression, and integrin α2-negative cell sorting. Graft-organizing function was tested by wrapping biphasic calcium phosphate (BCP) granules with SMCS and evaluating ectopic hard tissue formation in mice.

**Results:**

At a defined seeding density, DPSCs reached confluence at the D8 critical point and generated harvestable SMCS by D10, whereas BMSCs and UCSCs did not form SMCS. The puncture-force assay quantified a significantly higher peak puncture force in SMCS than in BMSC monolayers, converting subjective handleability into a quantitative mechanical measure. The D8–D10 2D-to-3D transition was marked by the COL1 Matrix Burst, dependence on TGF-β signaling, and integrin α2-dependent structural integrity. SMCS wrapped BCP granules into cohesive, cell-rich grafts before transplantation. In vivo, SMCS grafts generated significantly more new hard tissue than both DPSC and BMSC suspension grafts, demonstrating an effect beyond the DPSC source alone.

**Conclusions:**

This study defines DPSC self-multilayering as a distinct, density-gated, matrix-anchored process of self-organization that generates SMCS. A novel standardized puncture-force assay established SMCS as a mechanically quantifiable, surgically handleable cell sheet. By organizing BCP granules and enhancing in vivo hard tissue formation, SMCS provides a practical regenerative graft for bone tissue engineering.

**Graphical Abstract:**

**Figure:**
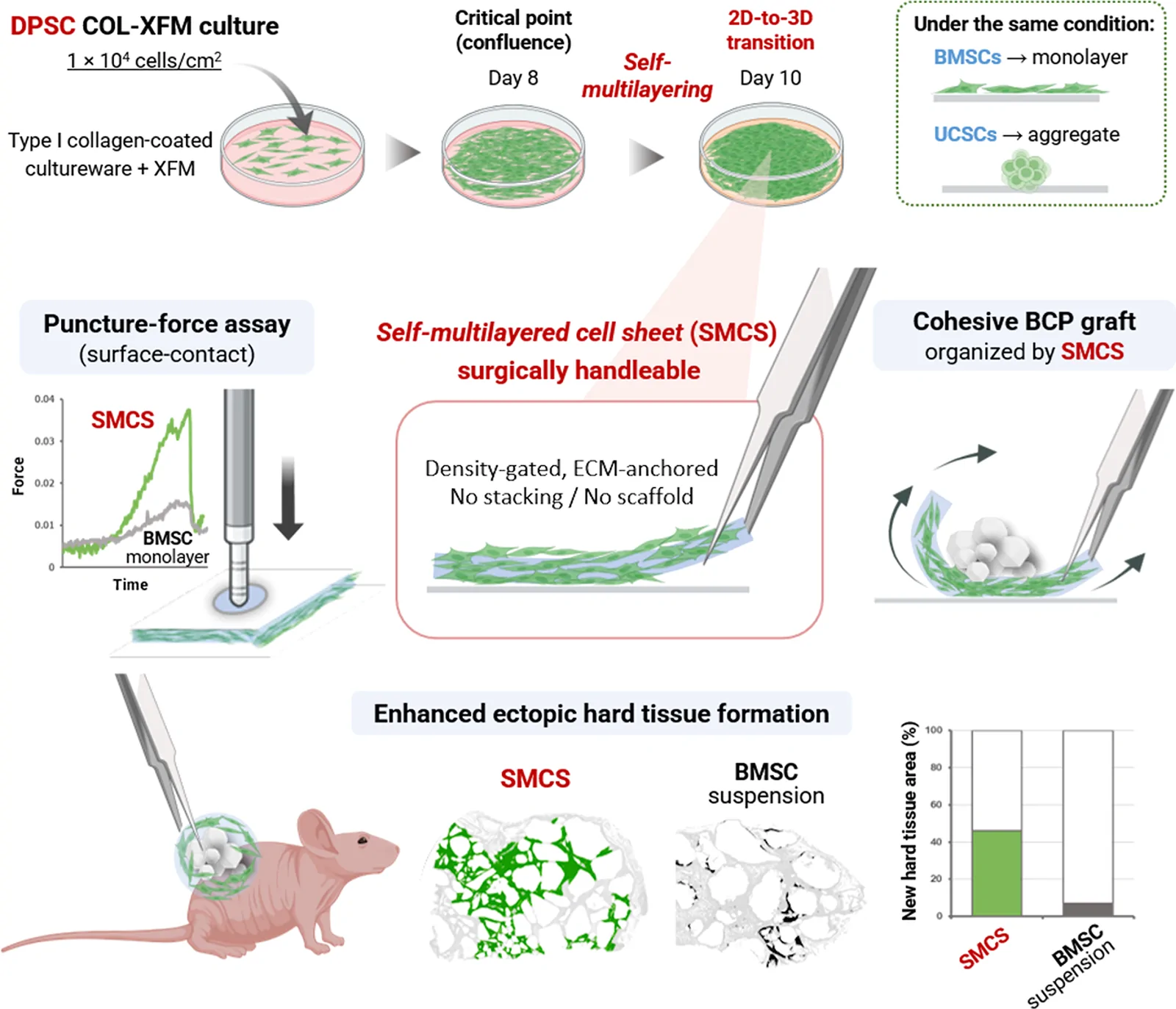

**Supplementary Information:**

The online version contains supplementary material available at https://doi.org/10.1186/s13287-026-05304-x.

## Background

Dental pulp stem cells (DPSCs) are an attractive human mesenchymal stromal cell (MSC) source for regenerative medicine. They possess stem cell properties and can be obtained from extracted teeth with relatively low invasiveness, expanded and banked for future therapeutic use [1–3]. In earlier studies, we established xenogeneic serum-free medium (XFM)-based methods for DPSC isolation, expansion, and cryopreservation [4]. We subsequently refined this approach using type I collagen-coated cultureware in XFM, designated COL-XFM culture. Under this condition, DPSCs formed viable multilayered structures through HIF-1α-dependent production of type I collagen (COL1) and COL1–integrin α2β1/α11β1 interactions [5]. However, these multilayers had not been examined as harvestable scaffold-free cell sheets.

Cell sheet technology has provided an important scaffold-free approach for regenerative medicine by preserving cell–cell and cell–extracellular matrix (ECM) connections [6–8]. However, conventional cell sheet approaches often require external procedures, such as temperature-responsive detachment, manual stacking, or additional supporting materials, to obtain transplantable tissue constructs [6, 7, 9]. For clinical translation, scaffold-free cell sheets should be generated reproducibly, harvested without tearing, and mechanically stable enough for surgical manipulation. Nevertheless, objectively quantifying the mechanical reliability of fragile MSC sheets remains challenging [10, 11]. This gap highlights the need for an objective assay that connects mechanical integrity with practical graft handleability.

Here, we found that overconfluent DPSC multilayers in COL-XFM culture could be detached with fine forceps as thick, handleable cell sheets. This observation led us to hypothesize that DPSCs can autonomously generate a mechanically stable scaffold-free tissue without manual stacking or exogenous scaffold support. We therefore define this structure as a DPSC-derived *self-multilayered cell sheet* (SMCS), a distinct scaffold-free tissue formed through autonomous self-multilayering of DPSCs.

In this study, we first optimized SMCS generation under COL-XFM culture. The resulting DPSC-optimized condition was used to compare DPSCs with bone marrow-derived and umbilical cord-derived MSCs (BMSCs and UCSCs, respectively). We established a puncture-force assay to quantify the mechanical integrity and handleability of fragile scaffold-free cell sheets. To define the day 8 (D8) to day 10 (D10) transition from a confluent two-dimensional (2D) monolayer to three-dimensional (3D) SMCS, we analyzed COL1 production dynamics, TGF-β signaling, and integrin α2-mediated matrix interaction. We further evaluated whether SMCS could organize biphasic calcium phosphate (BCP) granules and enhance ectopic hard tissue formation in vivo. Through these analyses, we aimed to define self-multilayering as a distinct, density-gated, ECM-anchored self-organizing process and to establish SMCS as a mechanically quantifiable regenerative graft for bone tissue engineering.

## Methods

### Cell sources and COL-XFM culture

DPSCs were obtained from dental pulp tissues of third molars extracted for therapeutic reasons from eight donors aged 21–37 years, as described previously [5]. UCSCs were isolated from umbilical cord tissues from eight donors aged 27–41 years and expanded under the same COL-XFM culture condition. BMSCs were recovered from cryopreserved stocks established in our previous study [12]. These MSCs were expanded under COL-XFM culture conditions. Briefly, cells were cultured on 60-mm culture dishes (Corning, NY, USA) coated with type I collagen (Cellmatrix Type I-C; Nitta Gelatin, Osaka, Japan) in XFM (PRIME-XV MSC Expansion XSFM; Irvine Scientific, Santa Ana, CA, USA) at 37 °C in a humidified atmosphere containing 4.7% CO₂. At 80% confluence, cells were detached with TrypLE Express Enzyme (Thermo Fisher Scientific, Waltham, MA, USA) and replated at 5 × 10³ cells/cm². Cells at passages 3–5 were used for all experiments.

### Growth curve analysis and evaluation of SMCS formation

Growth curve analysis was performed according to our previous method [5]. For seeding-density optimization, DPSCs were seeded at 0.5, 0.75, 1, 1.5, 2, or 4 × 10^4^ cells/cm² in 12-well plates and cultured for 12 days. To compare self-multilayering among MSC types, DPSCs, BMSCs, and UCSCs were seeded at 1 × 10^4^ cells/cm² and cultured for up to 12 days. Cell numbers were counted every 2 days in triplicate using a hemocytometer. Phase-contrast images were obtained at the indicated time points.

### Histological and TUNEL analyses of in vitro cultures

Cultures were fixed with 4% paraformaldehyde (PFA), embedded in optimal cutting temperature (OCT) compound, frozen, and serially cryosectioned at 12 μm using a cryostat. Sections were stained with hematoxylin and eosin (HE) or Masson’s trichrome (MT) according to standard protocols. Apoptotic cells were detected by terminal deoxynucleotidyl transferase-mediated dUTP nick-end labeling (TUNEL) staining according to the manufacturer’s instructions (Roche Diagnostics, Mannheim, Germany). Stained sections were examined using a BZ-X810 microscope (Keyence, Osaka, Japan). The thickness of SMCS and BMSC monolayers at D10 was measured on HE sections using BZ-X800 Analyzer software (Keyence). For each group, five sections from each of five samples were analyzed, and five points were measured per section.

### Immunofluorescence analysis of in vitro cultures

Immunofluorescence staining was performed as described previously [4]. Sections were incubated with primary antibodies against COL1 (1:1000; Cell Signaling Technology, Danvers, MA, USA), integrin α2 (1:50; Santa Cruz Biotechnology, Dallas, TX, USA), or integrin α11 (1:50; Santa Cruz Biotechnology), followed by Alexa Fluor-conjugated secondary antibodies (Thermo Fisher Scientific). Isotype-matched antibodies were used as negative controls. Stained sections were mounted with Fluoro-KEEPER Antifade Reagent containing DAPI (Nacalai Tesque, Kyoto, Japan) and examined using a confocal laser-scanning microscope (LSM 700; Carl Zeiss, Oberkochen, Germany).

### Standardized puncture-force assay for scaffold-free cell sheets

To quantitatively evaluate the mechanical integrity of fragile scaffold-free cell sheets, we established a puncture-force assay using a force–displacement measurement unit (FSA-0.5KE-5N; IMADA, Toyohashi, Japan). The measurement system was equipped with a digital force gauge (ZTA-2N; IMADA) with a load resolution of 0.001 N and a sampling rate of 2000 Hz. Because edge gripping can damage fragile scaffold-free cell sheets before the intended test region fails, we designed a surface-contact fixture consisting of a custom specimen stage and a matching cover plate, both with a 5-mm-diameter central aperture. D10 SMCS or BMSC monolayers were placed on the specimen stage and secured with the cover plate by surface contact around the aligned apertures, leaving the unsupported central region available for localized puncture testing. A 3-mm-diameter cylindrical probe was driven vertically at a constant speed of 0.5 mm/s using a motorized test stand. The maximum force immediately before rupture was recorded as the puncture force. Data acquisition and analysis were performed using Force Recorder Professional software (version 1.03; IMADA). For each donor, four independently prepared samples were tested per group.

### Western blotting

Western blotting was performed to analyze temporal changes in COL1 protein levels. DPSC and BMSC cultures were harvested every 2 days from D4 to D10 and lysed in lysis buffer containing 10 mM Tris–HCl (pH 7.4), 1 mM MgCl₂, and 0.1% Triton X-100. Protein concentrations were measured using a Qubit Protein Assay Kit and Qubit 4 Fluorometer (Thermo Fisher Scientific). A total of 10 µg of protein was separated by sodium dodecyl sulfate–polyacrylamide gel electrophoresis, transferred onto polyvinylidene fluoride membranes, and probed with primary antibodies against COL1A1 (1:1,000; Cell Signaling Technology) and GAPDH (1:2,000; GeneTex, Irvine, CA, USA). Membranes were then incubated with horseradish peroxidase (HRP)-conjugated goat anti-rabbit IgG and HRP-conjugated horse anti-mouse IgG secondary antibodies (both 1:2,000; Cell Signaling Technology). Immunoreactive bands were visualized using an iBright imaging system (Thermo Fisher Scientific). Band intensities were quantified using ImageJ software (version 1.52a; National Institutes of Health, Bethesda, MD, USA) and normalized to GAPDH.

### Flow cytometry and cell sorting

Flow cytometry was performed to analyze MSC surface markers and integrin expression. For MSC marker profiling, DPSC and BMSC cultures were analyzed from D4 to D10 using a Human Mesenchymal Stem Cell Verification Flow Kit (FMC020; R&D Systems, Minneapolis, MN, USA). Integrin expression was analyzed using antibodies against integrin α2 (CD49b; clone 1E6-C5, FITC-conjugated; BioLegend, San Diego, CA, USA) and integrin β1 (CD29; clone TS2/16, PE-conjugated; BioLegend), together with corresponding isotype-matched controls. Debris and doublets were excluded based on forward and side scatter gating. Data were acquired using a BD FACSMelody cell sorter (BD Biosciences, San Jose, CA, USA) and analyzed using FlowJo software (version 10.0; BD Biosciences). Positive populations were defined using these controls, and mean fluorescence intensity (MFI) was calculated under identical acquisition settings. For cell sorting, integrin α2-negative DPSCs were isolated using a BD FACSMelody cell sorter with the same gating strategy. Sorted cells were cultured under COL-XFM conditions, and their capacity to form SMCS was evaluated at D10.

### A-83-01 treatment

To inhibit TGF-β signaling during the D8–D10 transition, DPSC cultures were treated with A-83-01, a TGF-β type I receptor inhibitor (Selleck Chemicals, Houston, TX, USA). A-83-01 was added on D8 at final concentrations of 1, 10, or 20 µM, and cultures were analyzed on D10. DMSO-treated cultures served as vehicle controls. The effects of A-83-01 treatment were evaluated by phase-contrast microscopy, histological staining, cell counting, and flow cytometric analysis of integrin expression.

### In vitro osteogenic differentiation

SMCS and BMSC cultures were subjected to osteogenic induction for 14 days according to our previous method [4]. Briefly, cultures were maintained in α-modified minimal essential medium supplemented with 10% fetal bovine serum, dexamethasone, β-glycerophosphate, and L-ascorbate-2-phosphate. Control cultures were maintained in medium without osteogenic supplements. Mineralized deposits were evaluated by Alizarin Red S staining and 0.1% (w/v) xylenol orange fluorescence (FUJIFILM Wako Pure Chemical, Osaka, Japan), imaged using a BZ-X810 microscope (Keyence), and quantitatively analyzed using BZ-X800 Analyzer software (Keyence).

### Preparation of grafts with BCP granules

BCP granules composed of hydroxyapatite and β-tricalcium phosphate (Ceraform; Niterra, Nagoya, Japan) were used as the ceramic carrier. Grafts were prepared using BCP granules together with SMCS, BMSCs, or DPSCs. For SMCS grafts, a single SMCS generated in a 12-well plate and containing approximately 1 × 10⁶ cells was used to wrap 40 mg of BCP granules. For BMSC and DPSC suspension grafts, 1 × 10⁶ cells were mixed with 400 µL of collagen gel (Cellmatrix Type I-A; Nitta Gelatin) and 40 mg of BCP granules, as described previously [13]. Cell-free BCP/collagen gel grafts served as controls. Graft organization and forceps manipulation before transplantation were observed using a fluorescence stereomicroscope (Axio Zoom.V16; Carl Zeiss).

### In vivo transplantation and animal procedures

The animal experiments are reported in accordance with the ARRIVE 2.0 guidelines. Grafts were transplanted subcutaneously into the dorsal region of 6-week-old male nude mice (BALB/cAJcl-nu/nu; CLEA Japan, Tokyo, Japan). This ectopic model was selected to evaluate graft-mediated de novo hard tissue formation in a non-osseous environment [14]. The study included three cell-containing experimental groups—SMCS/BCP grafts, BMSC/BCP/collagen gel grafts, and DPSC/BCP/collagen gel grafts—and cell-free BCP/collagen gel grafts as negative controls. Each mouse received four grafts from the same experimental group to avoid potential cross-contamination among graft types. Five mice were used per group, resulting in a total of 20 mice and 80 transplanted grafts. Quantitative analysis was performed at the mouse level by averaging measurements from grafts within each mouse, and the individual mouse was considered the experimental unit. For transplantation surgery, mice were anaesthetized by intraperitoneal administration of a three-drug anaesthetic mixture containing medetomidine hydrochloride (0.3 mg/kg), midazolam (4 mg/kg), and butorphanol tartrate (5 mg/kg). After surgery, anaesthesia was reversed by intraperitoneal administration of atipamezole hydrochloride (3.0 mg/kg), an antagonist of medetomidine. Animals were monitored according to the approved protocol; no animals reached the predefined humane endpoints. At 16 weeks after transplantation, mice were euthanized by intraperitoneal administration of pentobarbital (200 mg/kg), and the grafts were then harvested.

### Histological and immunohistochemical analyses of transplanted grafts

Grafts were fixed with 4% PFA, decalcified, embedded in paraffin, and serially sectioned at 5 μm [15]. Sections were stained with HE or MT according to standard protocols. Immunohistochemical staining was performed as described previously [16] using antibodies against human-specific vimentin (1:10,000; Merck KGaA, Darmstadt, Germany), osteopontin (1:500, Abcam, Cambridge, UK), and osteocalcin (1:200; Santa Cruz Biotechnology). Human alveolar bone sections were stained using the same antibodies as reference tissue. Sections incubated with isotype control antibodies served as negative controls. Images were obtained using a BZ-X810 microscope (Keyence). Newly formed hard tissue area was quantified on HE sections using BZ-X800 Analyzer software (Keyence). For each graft, five randomly selected fields were analyzed from each of five sections.

### Statistical analysis

Data are presented as the mean ± standard deviation. Experiments were independently repeated using cells from at least four donors for each MSC type examined. For analyses using independently prepared samples, histological sections, or microscopic fields, the units of analysis are described in the corresponding Methods sections. Normality was assessed using the Shapiro–Wilk test. Statistical analyses were performed using IBM SPSS Statistics software (version 23.0; IBM Japan, Tokyo, Japan) and GraphPad Prism software (version 10.6.0; GraphPad Software, Boston, MA, USA). Two-group comparisons were analyzed using an unpaired Student’s *t*-test or Mann–Whitney *U* test. Multiple-group comparisons were analyzed using one-way analysis of variance (ANOVA) followed by Tukey’s post hoc test, or the Kruskal–Wallis test followed by Dunn’s post hoc test. Comparisons among four matched groups were performed using repeated-measures one-way ANOVA with the Greenhouse–Geisser correction, followed by Dunnett’s multiple comparisons test to compare each treatment group with the control group. A value of *P* < 0.05 was considered statistically significant. Significance levels are indicated in each figure legend.

## Results

### A defined seeding density enables robust DPSC self-multilayering

**Fig. 1:**
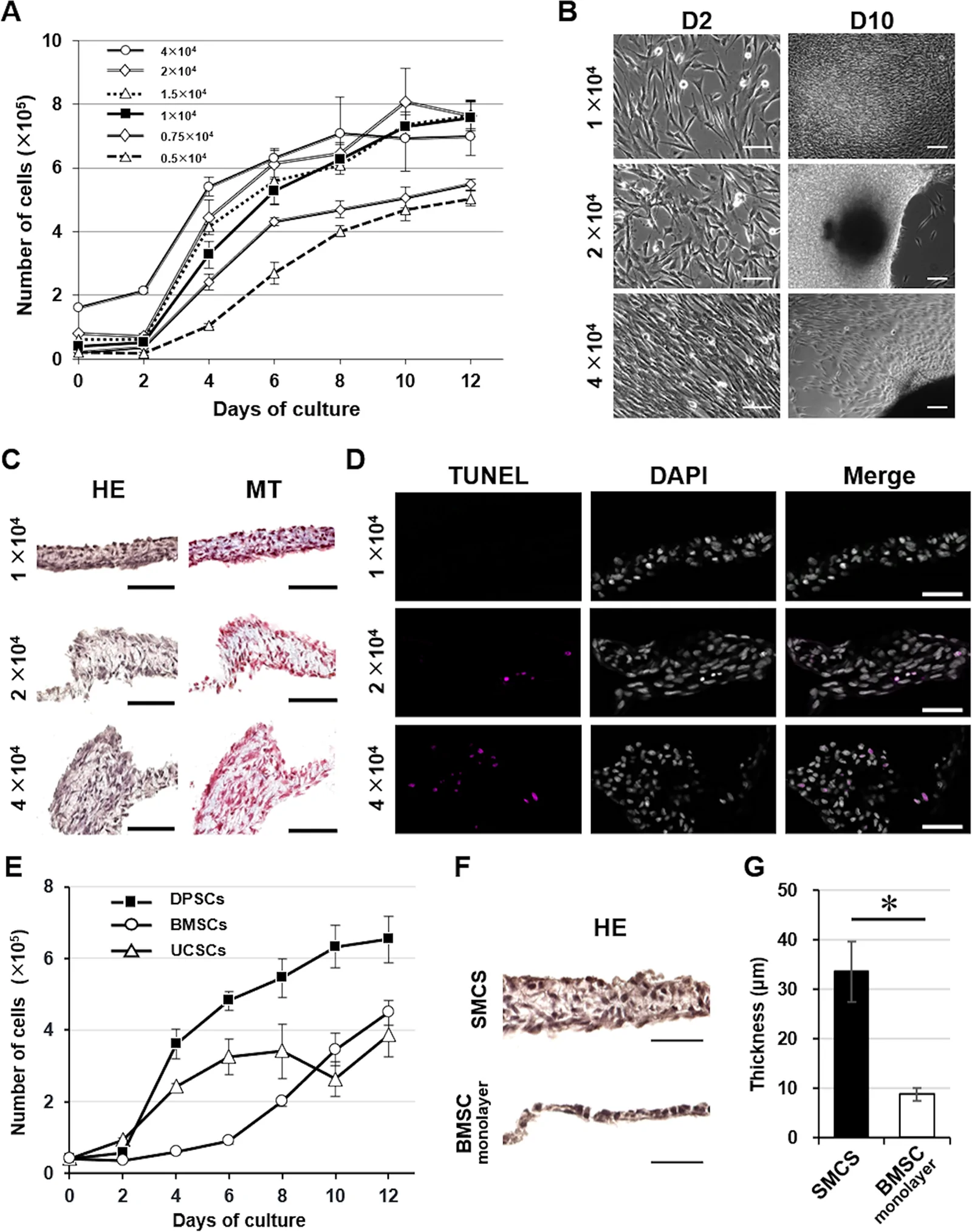
A defined COL-XFM condition reveals DPSC-specific SMCS formation among DPSCs, BMSCs, and UCSCs. **A** Growth curves of DPSCs seeded at 0.5, 0.75, 1, 1.5, 2, or 4 × 10⁴ cells/cm² and cultured for 12 days in COL-XFM. **B** Phase-contrast images of DPSC cultures at day 2 (D2) and day 10 (D10) after seeding at 1, 2, or 4 × 10⁴ cells/cm². **C** HE and MT staining of the corresponding D10 DPSC cultures. **D** TUNEL staining of the corresponding D10 DPSC cultures. Numbers in **A**–**D** indicate initial seeding densities (cells/cm²). **E** Growth curves of DPSCs, BMSCs, and UCSCs seeded at 1 × 10⁴ cells/cm² in COL-XFM. **F** HE sections of DPSC-derived *self-multilayered cell sheet* (SMCS) and BMSC monolayers at D10. **G** Quantification of D10 SMCS and BMSC monolayer thickness. Scale bars, 100 μm in **B**–**D** and 50 μm in **F**. *P < 0.01

To determine the optimal seeding condition for rapid and efficient SMCS generation, we first examined the effect of initial seeding density using DPSCs cultured in COL-XFM. Our previous COL-XFM study showed that DPSCs seeded at 0.5 × 10⁴ cells/cm² formed multilayered structures during prolonged overconfluent culture [5]. Based on this finding, we tested six seeding densities ranging from 0.5 × 10⁴ to 4 × 10⁴ cells/cm². Growth curve analysis revealed active proliferation in all groups up to D12 (Fig. 1A), although the structural outcomes at D10 differed markedly among seeding densities (Fig. 1B). Thus, higher initial seeding densities did not result in SMCS formation. Instead, a relatively low seeding density of 1 × 10⁴ cells/cm² emerged as the most favorable condition, yielding a continuous, thick 3D multilayered structure within 10 days of culture (Fig. 1C, D). In this group, cultures reached confluence by D8, marking a critical point after which self-multilayering became evident (Fig. 1A, C). By contrast, higher seeding densities, particularly 2 × 10⁴ and 4 × 10⁴ cells/cm², resulted in irregular aggregates with internal collapse and marked apoptotic cell death (Fig. 1B–D). Seeding densities close to the optimum (0.75 × 10⁴ and 1.5 × 10⁴ cells/cm²) yielded self-multilayered sheets that could be handled with forceps. However, the resulting sheets were thinner and less robust than those generated at 1 × 10⁴ cells/cm², indicating suboptimal conditions (Additional File 1: Supplementary Fig. 1).

### DPSCs, but not BMSCs or UCSCs, generate SMCS under the optimized COL-XFM condition

We next examined whether other clinically relevant MSCs, specifically BMSCs and UCSCs, exhibited similar self-multilayering under the DPSC-optimized COL-XFM condition (1 × 10⁴ cells/cm²). DPSCs proliferated steadily and generated thick, continuous SMCS, with a mean thickness of 33.0 ± 6.2 μm (Fig. 1E–G). In contrast, BMSCs showed slower growth and, after reaching confluence by D8, remained a thin confluent monolayer through D12, with no evidence of multilayer formation (Fig. 1E, F). The mean thickness of the BMSC monolayer was 9.0 ± 1.4 μm at D10 (Fig. 1G). UCSCs proliferated during the early phase of culture, but later underwent a transient reduction in cell number at D10 (Fig. 1E). This change was accompanied by detachment from the culture surface and the formation of irregular aggregates containing TUNEL-positive cells (Additional File 2: Supplementary Fig. 2). By contrast, both SMCS and the confluent BMSC monolayer were TUNEL-negative at D10 (Additional File 3: Supplementary Fig. 3). Thus, under this DPSC-optimized benchmark condition, BMSC cultures remained monolayers, and UCSC cultures detached and formed irregular aggregates.

Both DPSC and BMSC cultures retained a typical MSC marker profile (CD73, CD90, and CD105) during the culture period, as confirmed by flow cytometry (Additional File 4: Supplementary Table 1).

### SMCS is surgically handleable and mechanically quantifiable

**Fig. 2:**
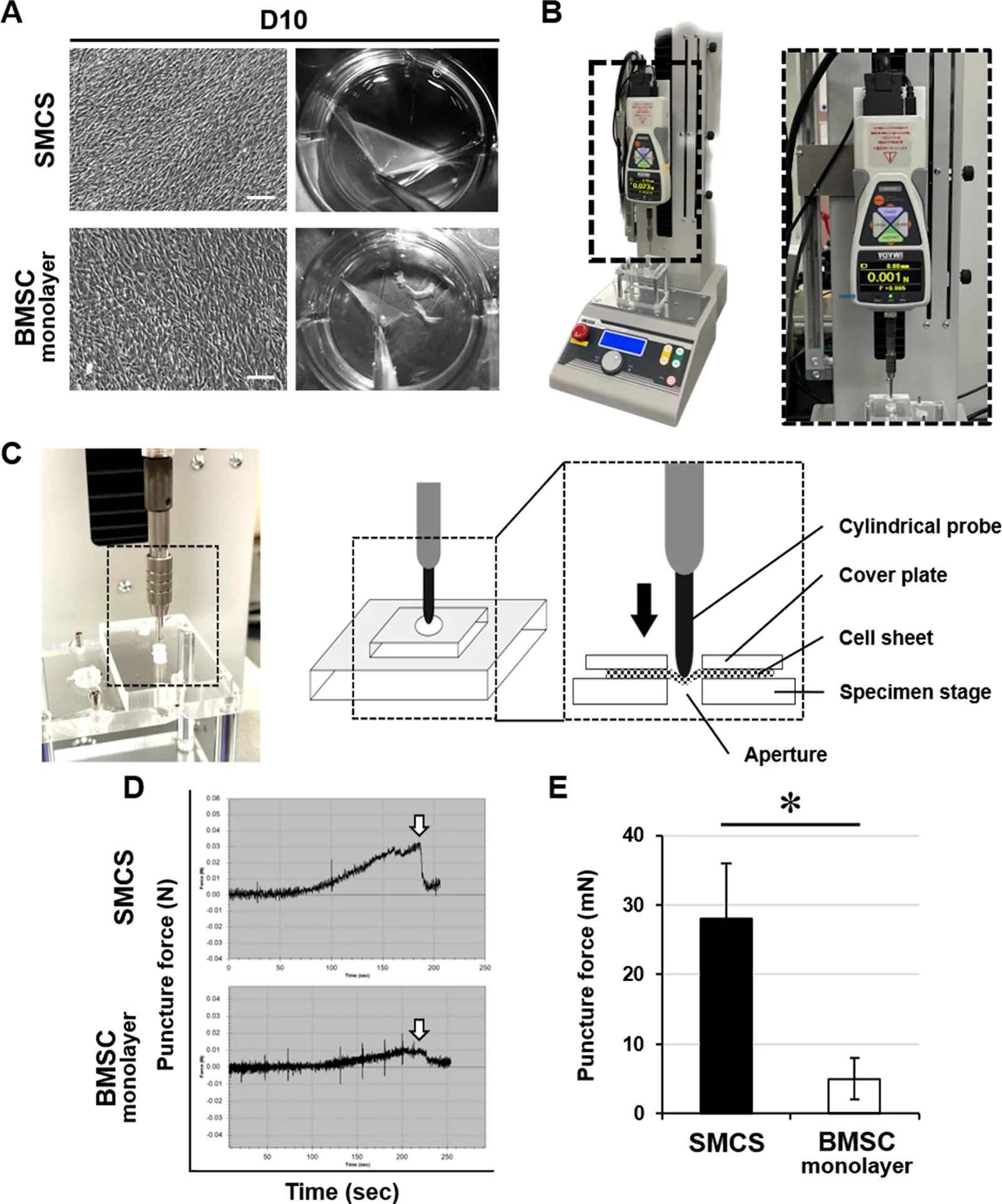
Development of a standardized puncture-force assay for scaffold-free cell sheets. **A** Phase-contrast and gross images of SMCS and BMSC cultures at D10. SMCS was harvested as a continuous sheet with fine forceps, whereas BMSC monolayers tore readily during forceps manipulation. Scale bars, 100 μm. **B** Overview of the force–displacement measurement unit used for puncture-force testing (left) and close-up view of the digital force gauge (right). **C** Photograph and schematic of the surface-contact puncture-force assay. A cell sheet was secured between a specimen stage and a matching cover plate, both with a central aperture. The unsupported central region was punctured with a cylindrical probe. The downward arrow indicates the direction of probe movement. **D** Representative force–time curves obtained during puncture testing of SMCS and BMSC monolayers. Arrows indicate the peak force immediately before sheet puncture. **E** Quantification of peak puncture force. *P < 0.01

To assess the mechanical properties and handleability of SMCS, we evaluated D10 cultures at the intended harvest time point. SMCS was harvested as a single, continuous sheet using fine forceps without tearing (Fig. 2A, Additional File 5: Supplementary Video 1). In contrast, BMSC cultures remained fragile confluent monolayers and tore readily during forceps manipulation (Fig. 2A, Additional File 6: Supplementary Video 2).

Force–displacement analysis revealed distinct mechanical profiles for SMCS and BMSC monolayers (Fig. 2B, C). The force increased progressively as the probe deformed the specimen, reached a peak force, and then rapidly decreased at the point of puncture (Fig. 2D). SMCS exhibited a significantly higher peak puncture force than BMSC monolayers (28 ± 8 mN vs. 5 ± 3 mN; Fig. 2E, *P* < 0.01). The gradual increase in force before puncture was consistent with the observed malleability of SMCS during forceps manipulation. The higher peak force reflected its greater rupture resistance (Fig. 2D).

### The COL1 Matrix Burst and sustained integrin α2 expression characterize DPSC self-multilayering

**Fig. 3:**
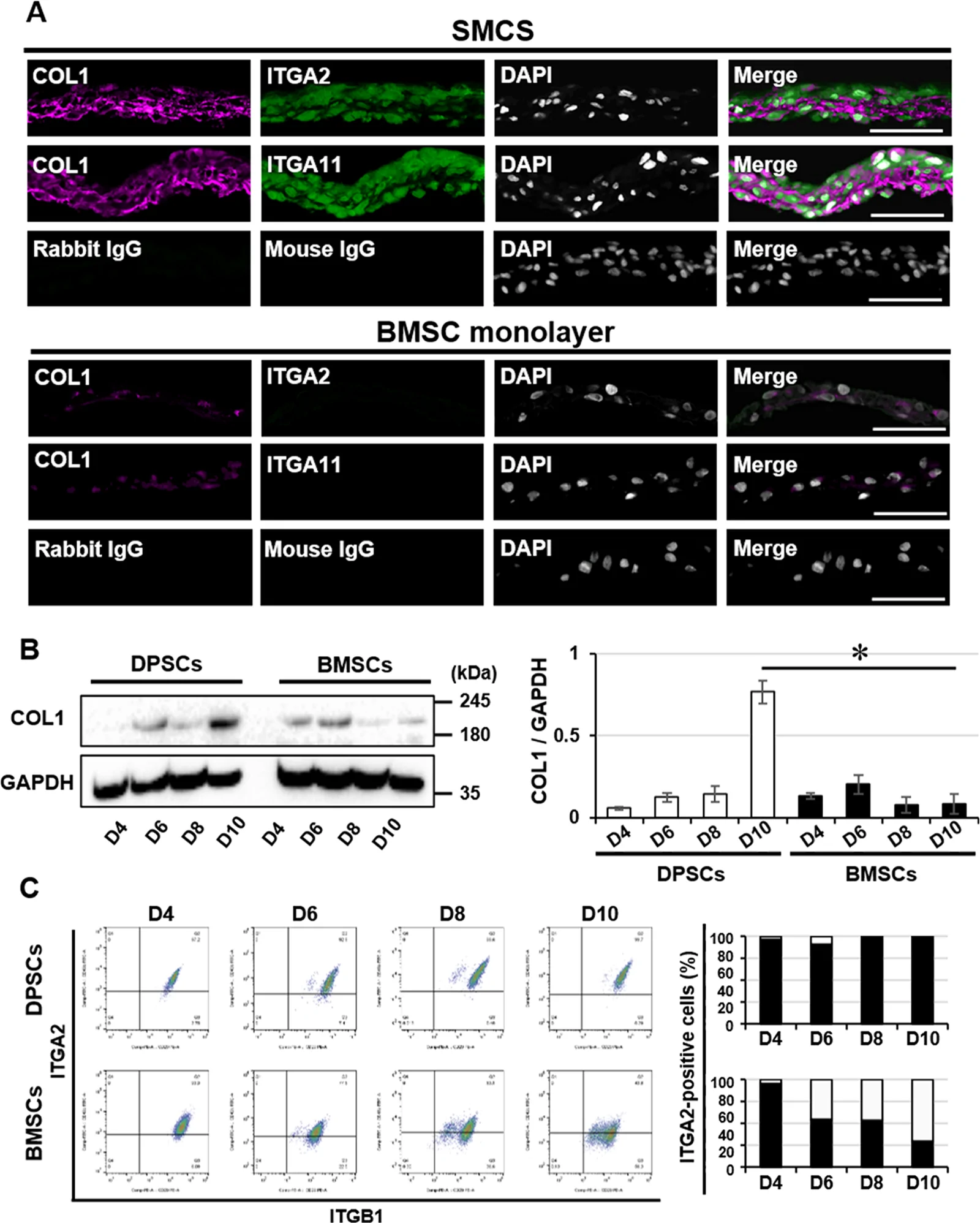
The COL1 Matrix Burst and sustained integrin α2 expression distinguish DPSC self-multilayering. **A** Immunofluorescence staining for COL1 with ITGA2 or ITGA11 in D10 SMCS and BMSC monolayer cultures. Rabbit IgG and mouse IgG were used as negative controls. Scale bars, 50 μm. **B** Western blot analysis of COL1 protein levels in DPSC and BMSC cultures from D4 to D10. Densitometric quantification of COL1 normalized to GAPDH. *P < 0.01. Uncropped full-length blots/gels are provided in Additional File 7: Supplementary Fig. 4. **C** Flow cytometric analysis of ITGA2 and ITGB1 expression in DPSC and BMSC cultures from D4 to D10, with quantification of ITGA2-positive cells. ITGA2, integrin α2; ITGA11, integrin α11; ITGB1, integrin β1

We next characterized COL1 and major COL1-binding integrins during SMCS formation. At D10, SMCS exhibited robust and uniform expression of COL1, integrin α2, and integrin α11 throughout the multilayered tissue, whereas BMSC monolayers had weak COL1 and negligible integrin α2/α11 expression (Fig. 3A).

Western blot analysis revealed that COL1 protein levels in DPSC cultures remained low through D8 and then increased markedly by D10 (Fig. 3B). We refer to this D8–D10 increase as the “COL1 Matrix Burst.” In contrast, COL1 levels in BMSCs remained low throughout the culture period (Fig. 3B). Flow cytometry confirmed that DPSCs retained an integrin α2-positive population at nearly 100% up to D10, whereas this population declined after D4 in BMSCs and was markedly reduced by D10 (Fig. 3C). Integrin β1 positivity remained stable in both cell types. These results identify the COL1 Matrix Burst during D8–D10 and sustained integrin α2 expression as defining molecular features of DPSC self-multilayering.

### TGF-β signaling and integrin α2 are required for the D8–D10 2D-to-3D transition during SMCS formation

**Fig. 4:**
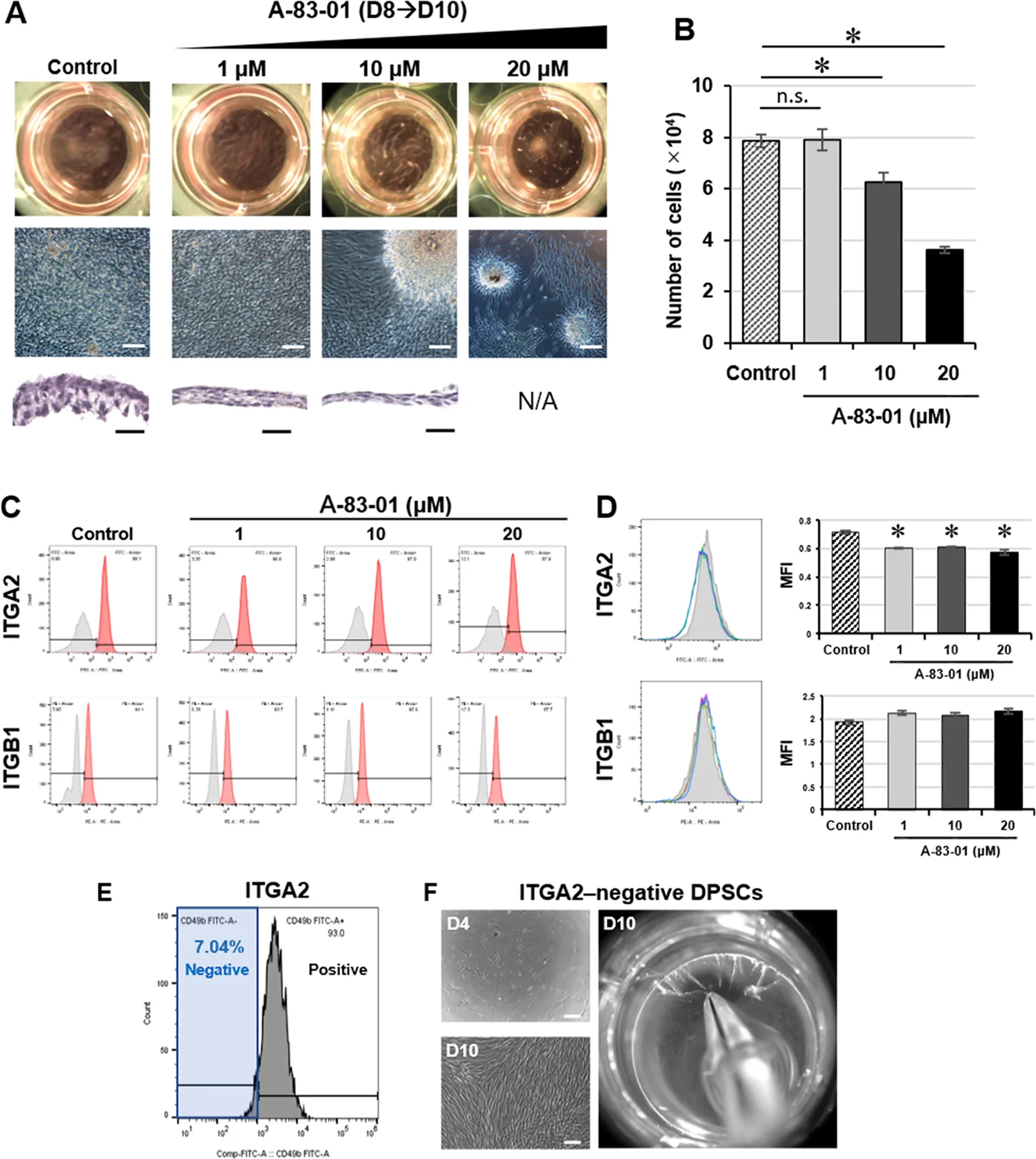
TGF-β signaling and integrin α2 control the D8–D10 2D-to-3D transition during SMCS formation. **A** Gross views, phase-contrast images, and HE sections of DPSC cultures treated with A-83-01 from D8 to D10. Control cultures formed a thick SMCS, whereas A-83-01 treatment progressively disrupted sheet formation. At 20 µM, severe aggregation prevented histological evaluation (N/A, not available). Scale bars, 100 μm for phase-contrast images and 50 μm for HE sections. **B** Quantification of cell number at D10 after A-83-01 treatment. *P < 0.01; n.s., not significant. **C** Flow cytometric histograms of ITGA2 and ITGB1 expression in control and A-83-01-treated DPSC cultures. Both markers were detected across all groups. **D** Overlay histograms and MFI quantification of ITGA2 and ITGB1. A-83-01 significantly reduced ITGA2 fluorescence intensity, whereas ITGB1 remained largely unchanged. *P < 0.01. **E** Sorting strategy for isolation of ITGA2-negative DPSCs. **F** Phase-contrast images of ITGA2-negative DPSCs at D4 and D10, and gross view during forceps manipulation at D10. ITGA2-negative DPSCs reached confluence but did not form a handleable SMCS. Scale bars, 100 μm. ITGA2, integrin α2; ITGB1, integrin β1; MFI, mean fluorescence intensity

To examine whether TGF-β signaling is required after the D8 critical point, we added A-83-01 to DPSC cultures at D8. Vehicle-treated cultures formed thick multilayered structures by D10, whereas A-83-01 treatment disrupted SMCS formation in a concentration-dependent manner (Fig. 4A). Treatment with 1 µM A-83-01 caused sheet thinning, 10 µM induced prominent aggregation with marked disruption of the sheet structure, and 20 µM caused severe aggregation with exposure of the culture surface, precluding histological evaluation. Cell yield at D10 was significantly reduced at higher concentrations of A-83-01 (Fig. 4B; *P* < 0.01). These data identify the D8–D10 interval as a critical window during which TGF-β signaling is required for the 2D-to-3D transition underlying SMCS formation.

We next examined integrin α2 expression during this 2D-to-3D transition. Flow cytometry detected integrin α2 and integrin β1 in all groups after A-83-01 treatment (Fig. 4C). However, A-83-01 treatment induced a leftward shift in integrin α2 fluorescence intensity and significantly reduced integrin α2 MFI (Fig. 4D; *P* < 0.01), whereas integrin β1 remained largely unchanged. To test the functional relevance of integrin α2, we isolated integrin α2-negative DPSCs by cell sorting (Fig. 4E). Although these cells reached confluence, they failed to form a handleable multilayered sheet and tore readily during forceps manipulation at D10 (Fig. 4F). These results indicate that adequate integrin α2 expression is required for the structural integrity and handleability of SMCS.

### SMCS organizes BCP granules into cohesive, cell-rich grafts and enhances ectopic hard tissue formation

As a supplementary assessment of osteogenic potential, SMCS and BMSC cultures were subjected to conventional in vitro osteogenic induction for 14 days. Both groups formed Alizarin red S-positive mineralized deposits, but SMCS cultures produced significantly larger mineralized nodules than BMSCs (Additional File 8: Supplementary Fig. 5).

**Fig. 5:**
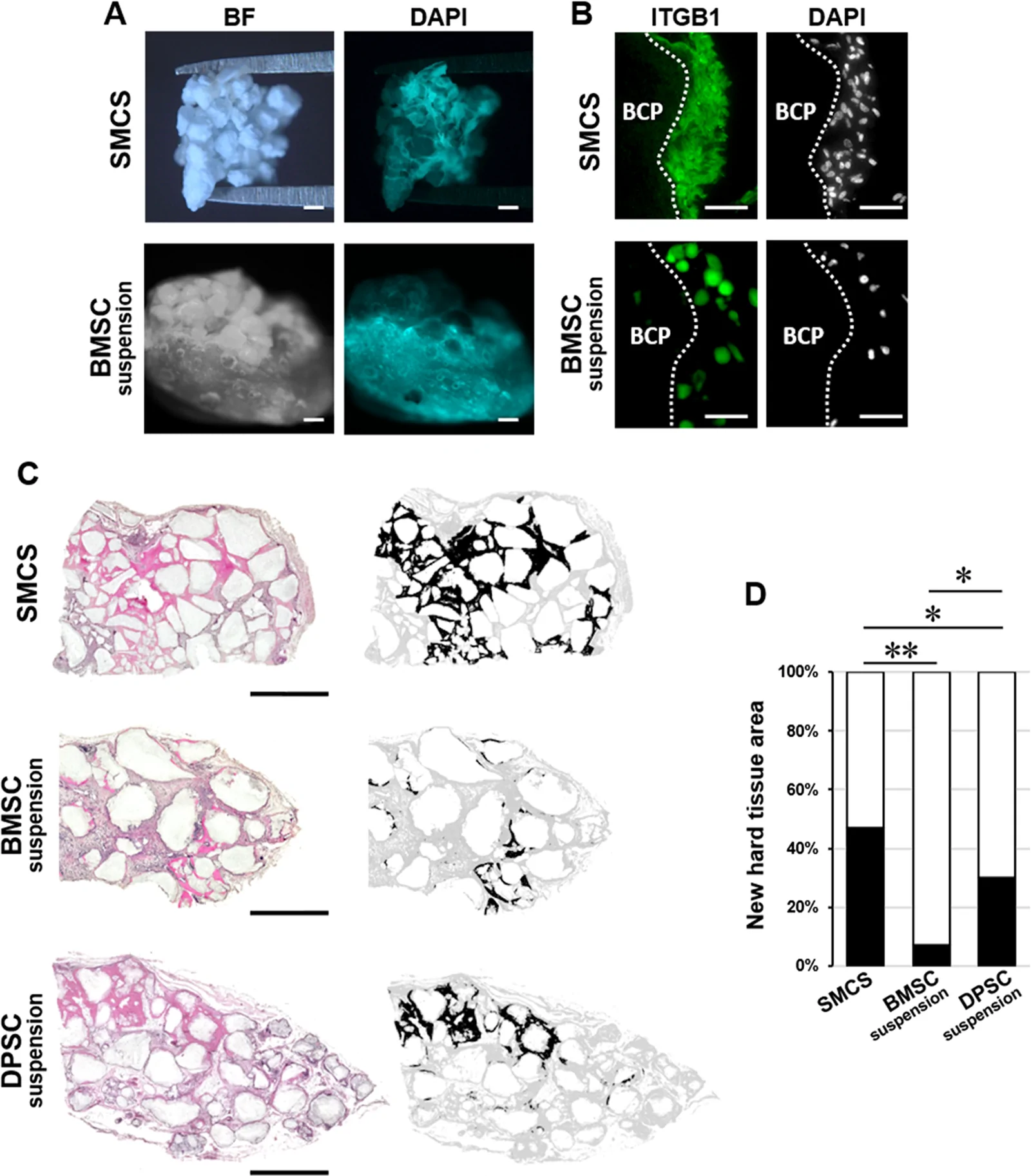
SMCS organizes BCP granules and enhances ectopic hard tissue formation. **A** Bright-field (BF) and DAPI fluorescence images of grafts with BCP granules before transplantation. BCP granules were either wrapped with a single SMCS or mixed with BMSC suspensions in collagen gel. In SMCS grafts, DAPI-positive cells broadly covered the BCP granules and formed a cohesive graft that could be grasped with forceps. In BMSC suspension grafts, BCP granules accumulated in the upper cell-poor region, whereas DAPI-positive cells were largely confined to the collagen gel below, with visible air bubbles in the gel. Scale bars, 1 mm. **B** Immunofluorescence images of the cell–BCP interface. In SMCS grafts, ITGB1-positive cells formed a continuous cellular layer along the BCP surface. In BMSC suspension grafts, round cells were scattered within the collagen gel and had limited attachment to BCP granules. Dotted lines indicate the BCP surface. Scale bars, 50 μm. **C** HE sections at 16 weeks after subcutaneous transplantation and corresponding images with newly formed hard tissue highlighted in black. Scale bars, 1 mm. **D** Quantification of the new hard tissue area in SMCS, BMSC suspension, and DPSC suspension grafts. *P < 0.05; **P < 0.01. BCP, biphasic calcium phosphate; BF, bright-field; ITGB1, integrin β1

To evaluate the relevance of SMCS to bone tissue engineering, we assessed in vivo hard tissue-forming capacity using an ectopic subcutaneous transplantation model in mice. BCP-based grafts were prepared either by wrapping BCP granules with a single SMCS or by mixing BCP granules with BMSC or DPSC suspensions in collagen gel. A single SMCS was sufficiently malleable to wrap BCP granules and generate a cohesive, cell-rich graft that could be grasped with forceps (Fig. 5A). In contrast, BMSC suspension grafts exhibited an uneven internal structure, with BCP granules accumulating in the upper cell-poor region and DAPI-positive cells largely confined to the collagen gel below (Fig. 5A). At the cell–BCP interface, SMCS grafts had a continuous layer of integrin β1-positive cells along the BCP surface (Fig. 5B). These cells showed spindle-shaped morphology and lay in close apposition to the granules. By contrast, BMSC suspension grafts contained round, scattered cells within the collagen gel, with limited spreading or direct attachment to BCP granules (Fig. 5B).

At 16 weeks after transplantation, SMCS grafts generated extensive new hard tissue within broad intergranular spaces between BCP granules (Fig. 5C). BMSC suspension grafts formed only limited hard tissue, whereas DPSC suspension grafts showed an intermediate phenotype (Fig. 5C). Histomorphometric analysis confirmed that the new hard tissue area was greatest in SMCS grafts, followed by DPSC and then BMSC suspension grafts, with significant differences among all three groups (Fig. 5D). Cell-free BCP/collagen gel grafts exhibited no evident hard tissue formation. These results demonstrate that SMCS enhanced ectopic hard tissue formation beyond the effect of the DPSC source alone.

### Newly formed hard tissue in SMCS grafts exhibits osteogenic features and donor-cell contribution

**Fig. 6:**
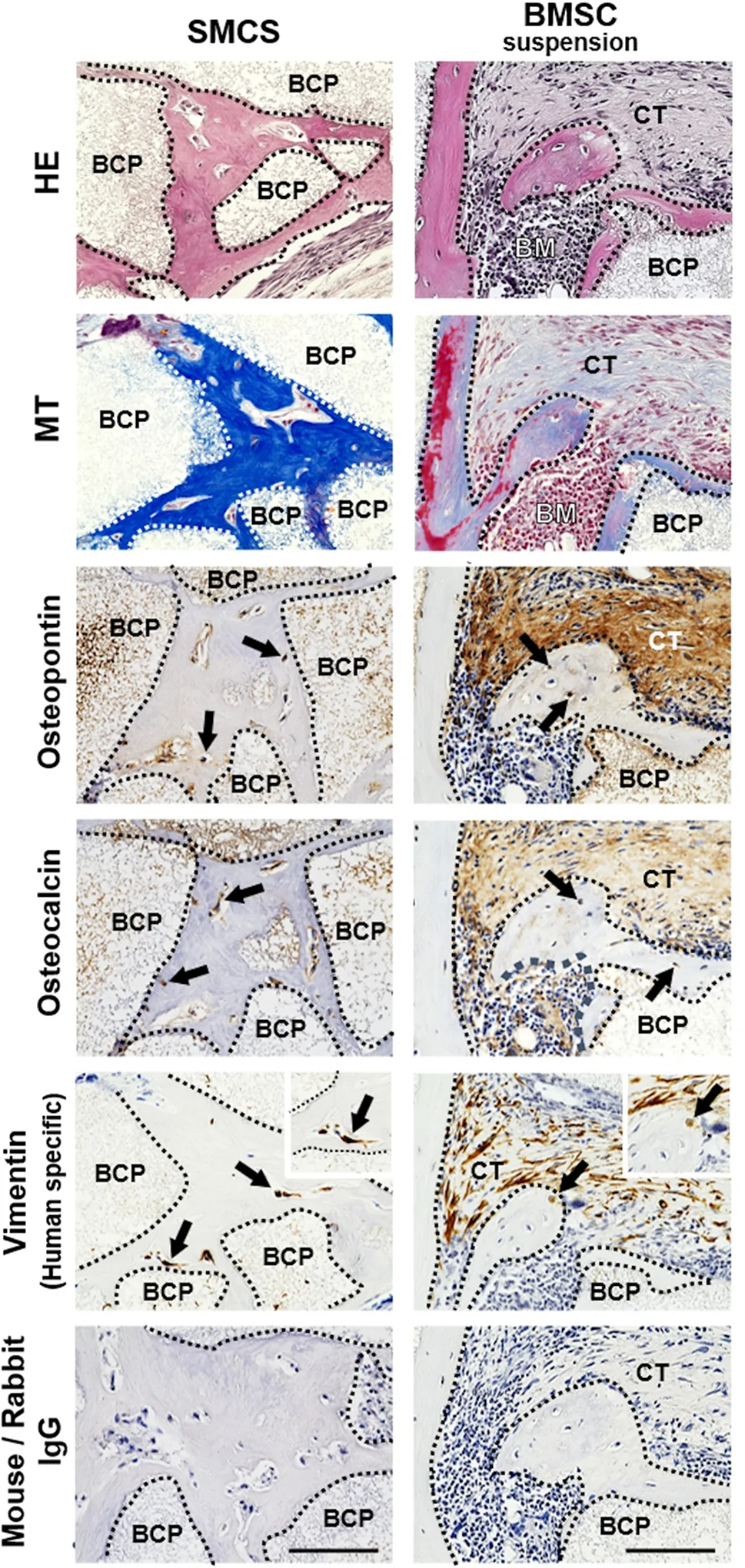
Osteogenic phenotype and donor-cell contribution in newly formed hard tissue of SMCS grafts. HE, MT, and immunohistochemical staining of SMCS and BMSC suspension grafts at 16 weeks after subcutaneous transplantation. In SMCS grafts, newly formed hard tissue occupied the spaces between BCP granules and contained embedded osteocyte-like cells. Osteopontin and osteocalcin immunoreactivity was detected in the newly formed hard tissue, including embedded osteocyte-like cells (arrows). Human-specific vimentin-positive embedded cells were also observed in this region. In BMSC suspension grafts, hard tissue formation was limited and accompanied by connective tissue (CT) and bone marrow-like tissue (BM). Mouse and rabbit IgG controls showed no specific staining. Dotted lines indicate the BCP surface. Scale bars, 100 μm. BCP, biphasic calcium phosphate

We then characterized the newly formed hard tissue and donor-cell contribution. In SMCS grafts, newly formed hard tissue occupied the spaces between BCP granules and contained embedded osteocyte-like cells (Fig. 6). Immunostaining for osteopontin and osteocalcin indicated an osteogenic phenotype in the newly formed hard tissue. Human-specific vimentin further demonstrated donor-cell contribution within this tissue. The same antibodies also stained human alveolar bone (Additional File 9: Supplementary Fig. 6). In BMSC suspension grafts, osteopontin- and osteocalcin-positive hard tissue was also observed, but the amount was limited and accompanied by abundant connective tissue and bone marrow-like tissue. Human-specific vimentin-positive cells were detected in both newly formed hard tissue and surrounding connective tissue (Fig. 6).

Together, these results demonstrate that SMCS generated larger, more continuous osteogenic hard tissue containing donor cells between BCP granules. This observation is consistent with the improved cell distribution and cell–BCP interface observed in SMCS grafts before transplantation.

## Discussion

Our previous COL-XFM study showed that DPSCs seeded at a conventional expansion density could form multilayered structures during prolonged overconfluent culture [5]. The present study refined this observation into a defined SMCS-forming condition, in which DPSCs reached a D8 critical point and generated harvestable SMCS by D10. SMCS formation therefore reflected an appropriate density-dependent 2D-to-3D transition rather than nonspecific overconfluence or excessive cell seeding. Consistent with this interpretation, higher initial seeding densities did not promote SMCS formation but instead disrupted organized sheet development, leading to disorganized aggregation and cell death. Once defined, this DPSC-optimized condition provided a clinically oriented benchmark for rapid and efficient SMCS generation. Under this condition, DPSCs generated harvestable SMCS within 10 days from an initial seeding density of 1 × 10⁴ cells/cm². By contrast, neither BMSCs nor UCSCs formed SMCS. This benchmark therefore emphasizes the practical value of obtaining harvestable SMCS within a short culture period from a relatively low initial seeding density.

To our knowledge, this is the first report of a puncture-force assay for scaffold-free cell sheets. Previous tensile testing of cell sheets required specialized fixtures to avoid clamping, piercing, or drying-related damage [10]. In contrast, our puncture-force assay held cell sheets by surface contact between an apertured stage and a matching cover plate, enabling simple, localized rupture-force measurement without edge gripping. This surface-contact setup minimized unintended sheet damage and converted the subjective impression of handleability into a quantitative measure of rupture resistance. Thus, the novel puncture-force assay adds a mechanical dimension to cell sheet assessment beyond thickness and gross morphology. It also provides a practical pre-implantation measure of surgical handleability for fragile scaffold-free cell sheets.

Mechanistically, the present findings extend the COL1–integrin axis from survival of overconfluent DPSC multilayers to the formation of harvestable SMCS. Our previous work demonstrated that this HIF-1α-dependent axis contributes to survival and stemness maintenance in overconfluent COL-XFM cultures [5]. Here, we show that the same axis, with integrin α2 as a central anchoring receptor, also underlies the D8–D10 transition that gives rise to SMCS. During this critical window, DPSCs underwent the COL1 Matrix Burst and maintained integrin α2 expression. TGF-β inhibition during this window disrupted SMCS formation, indicating that TGF-β signaling is required during the D8–D10 transition. Integrin α2-negative DPSCs also failed to form a handleable multilayered sheet. Thus, COL1–integrin interactions are not limited to maintaining overconfluent DPSC multilayers. In SMCS formation, they anchor the D8–D10 2D-to-3D transition and stabilize the emerging multilayered tissue. 

From the perspective of cell sheet engineering, conventional cell sheet approaches generally require external procedures, such as temperature-responsive detachment, manual stacking, or additional supporting materials, to obtain transplantable tissue constructs [6, 7, 9]. SMCS differs from these approaches because it is generated when a confluent 2D DPSC monolayer passes a density-dependent threshold and autonomously matures into a mechanically stable 3D multilayered tissue. We therefore propose self-multilayering as a density-gated, ECM-anchored mechanism of self-organization [17]. In this process, confluence acts as the spatial trigger, and the COL1 Matrix Burst provides the structural matrix. Integrin α2-mediated interaction with this COL1-rich matrix then stabilizes the emerging 3D tissue. Recent work using C2C12 myoblast sheets has also shown that confluent monolayers can undergo self-organized multilayering through defect-localized cell accumulation and ECM-mediated stabilization [18]. In the present study, SMCS formation is defined as density-gated, ECM-anchored self-multilayering rather than passive overgrowth. 

From the perspective of bone tissue engineering, ectopic subcutaneous transplantation has been widely used to evaluate the in vivo bone-forming capacity of DPSCs combined with scaffold materials [19]. We adapted the MSC/BCP subcutaneous transplantation model from our previous work [13] to evaluate SMCS as a tissue-organizing unit. In conventional cell-suspension grafts, cells and BCP granules are combined within a carrier material such as collagen gel. However, their pre-implantation organization is often assumed rather than directly examined. Here, fluorescence and histological analyses revealed that this format did not ensure uniform cell–material contact or structural cohesion before implantation. In contrast, a single SMCS wrapped the BCP granules as a continuous self-multilayered tissue, creating a cohesive, handleable graft with a cell-rich BCP interface. The intermediate outcome of DPSC suspension grafts indicates that enhanced hard tissue formation cannot be attributed to the DPSC source alone, but instead depends on pre-implantation SMCS organization around BCP granules. The immunostaining results further indicate that SMCS grafts generated osteogenic hard tissue containing donor-derived cells. Together, these in vivo findings establish that SMCS is not only harvestable but also functions as a tissue-organizing graft that creates a bone-regenerative microenvironment around BCP granules.

This study has several limitations. Because the detailed formulation of the commercially available XFM is not disclosed, the medium-derived factor(s) that initiate the D8–D10 2D-to-3D transition and COL1 Matrix Burst remain unidentified. In addition, the puncture-force assay quantified rupture resistance as one mechanical component of surgical handleability, but it does not capture all aspects of practical handling during graft preparation and implantation. Finally, although the MSC/BCP subcutaneous transplantation model remains ectopic, it provides a stringent test of graft-mediated hard tissue formation because hard tissue must arise de novo in a non-osseous environment [14]. Clinically oriented orthotopic bone-defect studies will nevertheless be required to evaluate the bone-regenerative capacity of SMCS-based grafts.

Overall, this study positions SMCS as a DPSC-derived scaffold-free tissue characterized by three features: autonomous self-multilayering, measurable mechanical integrity, and organization of BCP granules into cohesive grafts. These findings therefore distinguish SMCS from conventional cell sheets and identify self-multilayering as a density-gated, ECM-anchored self-organizing process in scaffold-free tissue engineering.

## Conclusions

This study establishes DPSC-derived SMCS as a scaffold-free 3D tissue generated through density-gated, ECM-anchored self-multilayering. A novel standardized puncture-force assay demonstrated that SMCS is mechanically quantifiable and surgically handleable. By organizing BCP granules into cohesive grafts and enhancing ectopic hard tissue formation, SMCS provides a practical regenerative graft for bone tissue engineering. 

## Supplementary Information

Below is the link to the electronic supplementary material.

Supplementary Material 1

Supplementary Material 2

Supplementary Material 3

## Data Availability

All data generated or analyzed during this study are included in this published article and its supplementary information files.

## Abbreviations

2D: Two-dimensional
3D: Three-dimensional
BCP: Biphasic calcium phosphate
BMSC: Bone marrow-derived mesenchymal stromal cell
COL1: Type I collagen
COL-XFM: Type I collagen-coated cultureware in xenogeneic serum-free medium
D: Day
DPSC: Dental pulp stem cell
ECM: Extracellular matrix
HE: Hematoxylin and eosin
ITGA2: Integrin α2
ITGA11: Integrin α11
ITGB1: Integrin β1
MFI: Mean fluorescence intensity
MSC: Mesenchymal stromal cell
SMCS: Self-multilayered cell sheet
TGF-β: Transforming growth factor-β
TUNEL: Terminal deoxynucleotidyl transferase-mediated dUTP nick-end labeling
UCSC: Umbilical cord-derived mesenchymal stromal cell
XFM: Xenogeneic serum-free medium
