# Supplementary material for: Self-multilayered cell sheet generated by dental pulp stem cells provides a mechanically quantifiable regenerative graft for bone tissue engineering
Source: Stem Cell Res Ther. 2026 Sep 22;17:405. doi: 10.1186/s13287-026-05304-x (PMC13733114; doi:10.1186/s13287-026-05304-x)
Supplement: Supplementary file 1 [file 13287_2026_5304_MOESM3_ESM.docx]

**Additional File 1: Supplementary Figure 1**

**Suboptimal self-multilayering at seeding densities close to the optimum.**

**A** Phase-contrast images and gross views during forceps manipulation of DPSC cultures seeded at 0.75 × 10⁴ or 1.5 × 10⁴ cells/cm². **B** HE and MT staining of the corresponding cultures. Both conditions yielded *self-multilayered cell sheets* that could be handled with forceps, but the sheets were thinner and less robust than those formed at 1 × 10⁴ cells/cm². Scale bars, 100 μm.

**Additional File 2: Supplementary Figure 2**

**Detachment and aggregate formation in UCSC cultures under the DPSC-optimized condition.**

**A** Gross image of a detached UCSC aggregate in a culture well (arrow and inset) and phase-contrast image of the culture surface. **B** TUNEL, DAPI, and merged images of a UCSC aggregate. TUNEL-positive cells were detected within the aggregate. Scale bars, 100 μm.

**Additional File 3: Supplementary Figure 3**

**TUNEL staining of D10 SMCS and confluent BMSC monolayer.**

Both structures were TUNEL-negative. Scale bars, 100 μm.

**Additional File 4: Supplementary Table 1**

**Additional File 4: Supplementary Table 1**

**Flow cytometric analysis of MSC marker expression during culture.**

Percentage expression of CD90, CD73, CD105, and negative markers in DPSC and BMSC cultures from D4 to D10. Negative markers were CD45, CD34, CD11b, CD79A, and HLA-DR.

**Additional File 5: Supplementary Video 1**

**Forceps manipulation of D10 SMCS.**

D10 SMCS was harvested and manipulated as a continuous sheet with fine forceps.

**Additional File 6: Supplementary Video 2**

**Forceps manipulation of the D10 BMSC monolayer.**

The D10 BMSC monolayer tore readily during forceps manipulation.

**Additional File 7: Supplementary Figure 4**

**Additional File 7: Supplementary Figure 4**

Uncropped full-length blots/gels corresponding to Fig. 3B. Boxed regions indicate the areas cropped for the representative COL1 and GAPDH blots shown in Fig. 3B.

**Additional File 8: Supplementary Figure 5**

**Additional File 8: Supplementary Figure 5**

**In vitro mineralized nodule formation after osteogenic induction.**

**A** Alizarin red S (ALZ) staining, xylenol orange fluorescence, and HE staining of SMCS and BMSC monolayer cultures after 14 days of osteogenic induction. Both groups formed ALZ-positive mineralized deposits. Larger nodular mineralized structures were observed in SMCS cultures. Insets show control cultures without osteogenic supplements. Arrows indicate mineralized nodules in HE sections of SMCS cultures. Scale bars, 100 μm. **B** Quantification of mineralized nodule size. The mean nodule area was significantly greater in SMCS cultures than in BMSC monolayer cultures. **P* < 0.05.

**Additional File 9: Supplementary Figure 6**

**Additional File 9: Supplementary Figure 6**

**Immunohistochemical staining of native human alveolar bone.**
Human alveolar bone was stained with the same antibodies against human-specific vimentin, osteopontin, and osteocalcin as those used for graft sections. Mouse and rabbit IgG served as negative controls. Arrows indicate immunopositive cells within native alveolar bone. Scale bar, 100 μm.
